# 3-Methyl-2-nitro­aniline

**DOI:** 10.1107/S2414314626008394

**Published:** 2026-08-20

**Authors:** Fariha A. Bushra, Marcus R. Bond

**Affiliations:** a Department of Chemistry and Mathematics Southeast Missouri State University,Cape Girardeau MO 63701 USA; University of Aberdeen, United Kingdom

**Keywords:** crystal structure, supra­molecular helix, aniline

## Abstract

The structure of the title compound, C_7_H_8_N_2_O_2_, is reported and completes structural characterization for the family of methyl-2-nitro­aniline isomers. Bond lengths and angles conform to reported values in other methyl-2-nitro­aniline structures, except for the large angle [36.82 (4)°] formed between the phenyl ring and nitro group mean planes. This large angle is due to steric crowding by the neighboring methyl group, a result that is confirmed by an *ab initio* geometry optimization *in vacuo* that yields a similar inter­planar angle (38.28°). In the extended structure, the mol­ecules form inversion-related pairs that belong to neighboring supra­molecular spiral stacks parallel to *c*. The stacks form around a 4_1_ axis with N—H⋯N hydrogen bonding between mol­ecules related by −1/4 of a turn at the core and peripheral N—H⋯O hydrogen bonds to mol­ecules related by +3/4 of a turn.

## Abstract 2

The structure of the title compound completes structural characterization for the family of methyl-2-nitro­aniline isomers. Bond lengths and angles conform to reported values in other methyl-2-nitro­aniline structures, except for the large angle [36.82 (4)°] formed between the phenyl ring and nitro group mean planes, which is due to steric crowding by the neighboring methyl group

## Graphical abstract

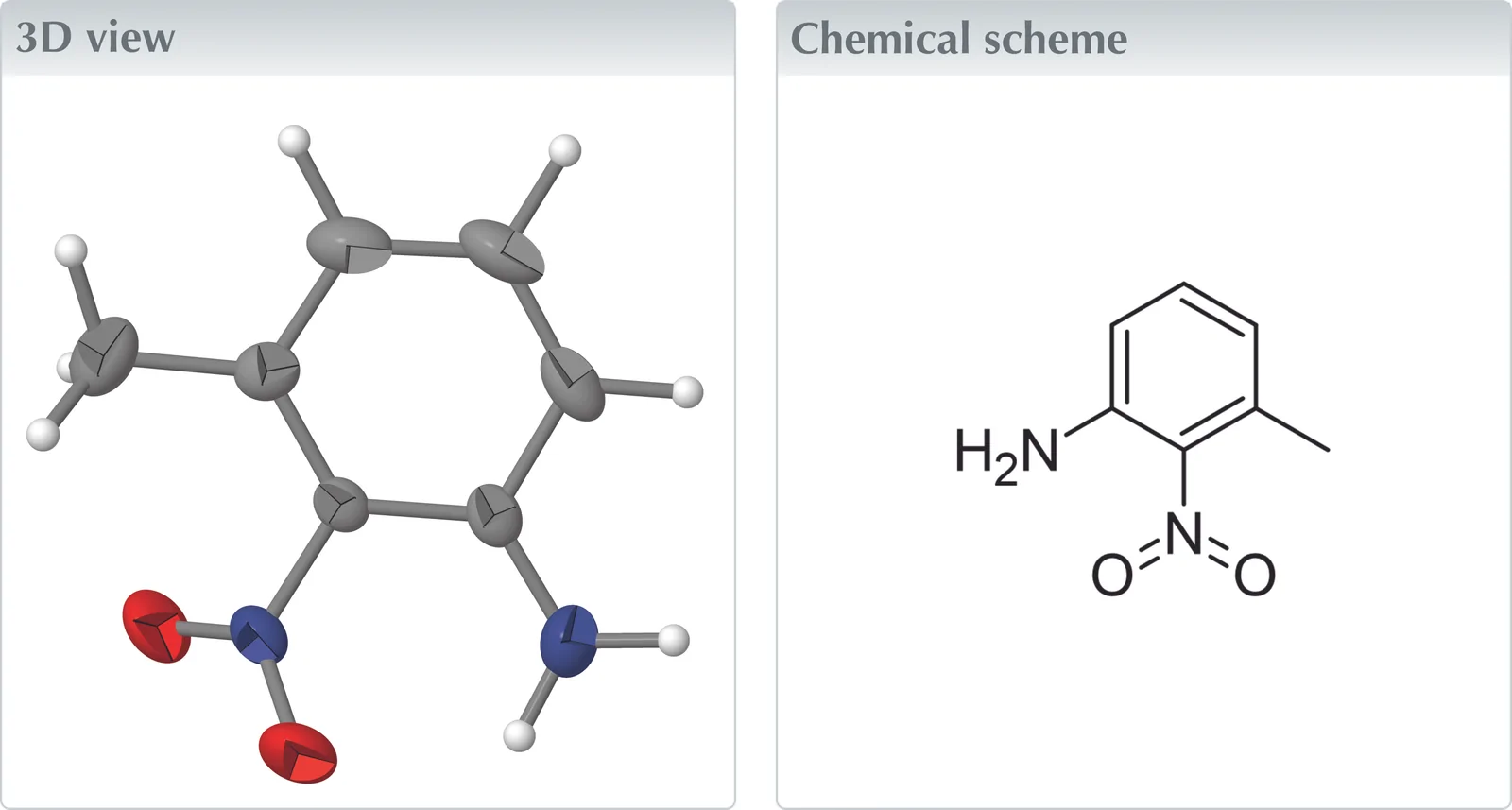

## Structure description

**Figure 1:**
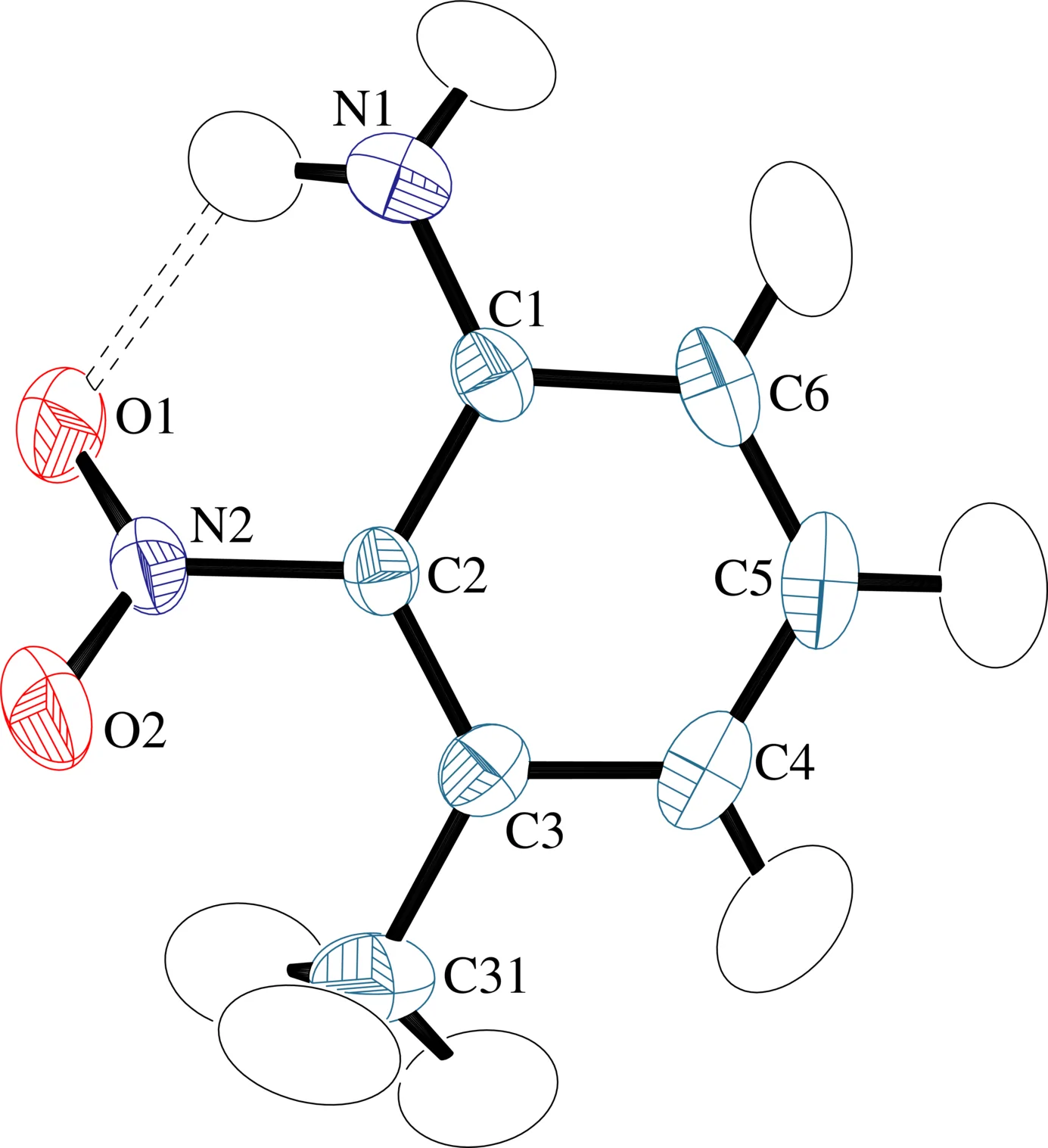
Displacement ellipsoid plot of (**I**) at the 50% level with labels for all non-H atoms. The intra­molecular hydrogen bond is indicated by a double-dashed line.

**Figure 2:**
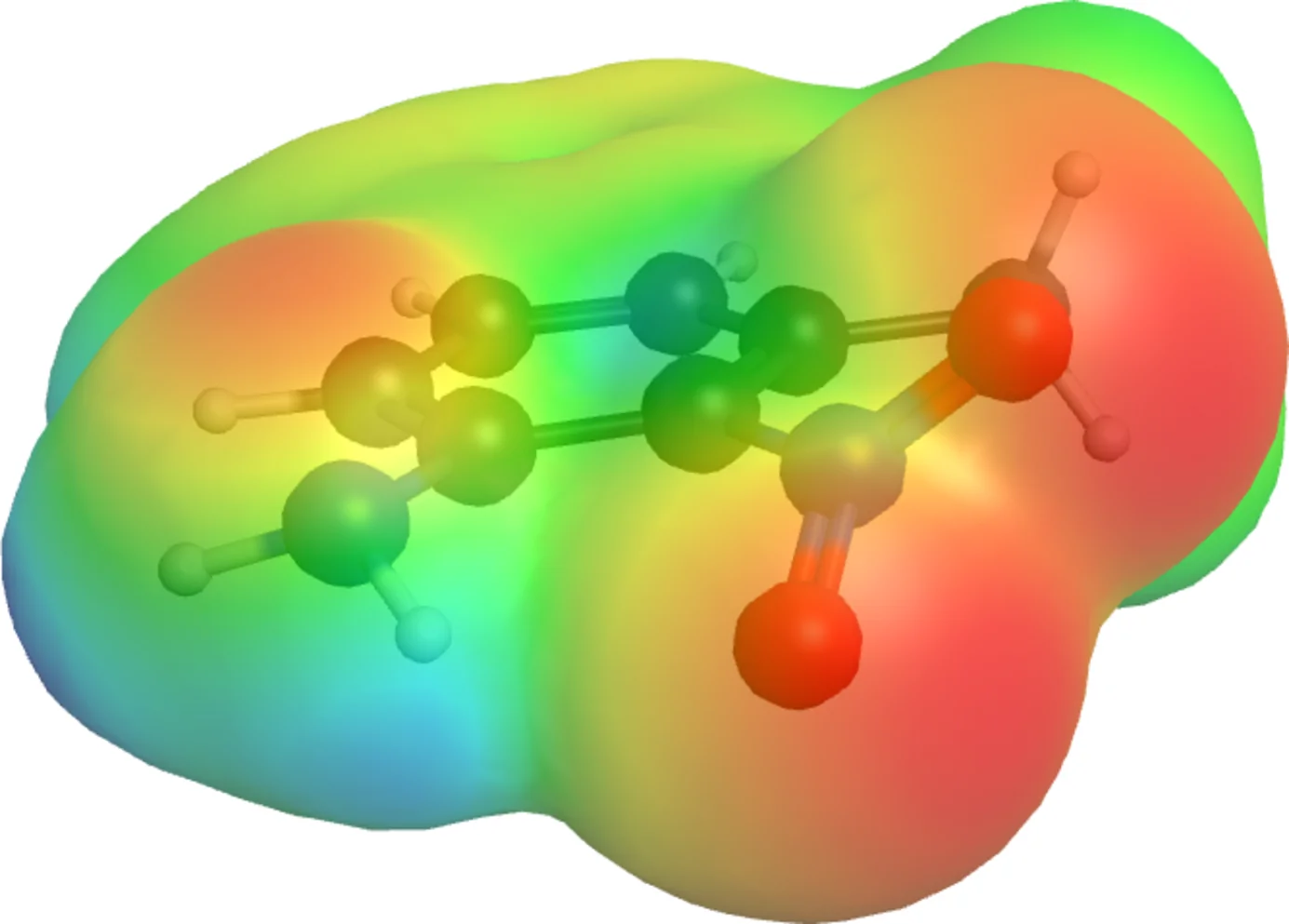
Electrostatic potential plot of the mol­ecular geometry of (**I**) obtained from an *ab initio* geometry optimization. Regions of negative charge accumulation are colored red while regions of positive charge accumulation are colored blue. Atoms are drawn as spheres of arbitrary radii.

The title compound, C_7_H_8_N_2_O_2_ (**I**), is the only methyl-2-nitro­aniline (M2NA) isomer with an unreported crystal structure. The geometric parameters of (**I**) (Fig. 1 ▸) conform to expected values and to the bond length and angle analysis conducted in our prior report of the structure of the 5-methyl isomer [Samson *et al.*, 2025 ▸; Cambridge Structural Database (CSD) refcode: ILADOS]. Specifically, the aniline group is almost planar [root-mean-square deviation (r.m.s.d.) = 0.056 Å from the C1/N1/H1*A*/H1*B* mean plane] with slight pyramidalization (N1 deviates by 0.097 Å from the mean plane while other atoms deviate by ∼0.03 Å on the opposite side). The nitro group is essentially planar (r.m.s.d. = 0.002 Å from the C2/N2/O1/O2 mean plane). The aniline group is nearly coplanar with the phenyl ring [dihedral angle = 4.9 (4)° between mean-plane normals] but the nitro group is not [36.82 (4)° between mean plane normals]. This large inter­planar angle results from steric crowding by the neighboring methyl group – in other M2NA structures this angle is less than 6°. This result is confirmed by an *ab initio* geometry optimization [6–31 G(*d*); *GAMESS* version 15 Jul 2024 (Schmidt *et al.*, 1993 ▸)] in which the value for this inter­planar angle (38.26°) is in close agreement. Of note, a corresponding DFT geometry optimization [B3LYP, 6–31 G(*d*)] underestimates this angle (22.36°) but nevertheless shows the nitro group plane at a substantial twist. An electrostatic potential plot of the *ab initio* optimization is presented in Fig. 2 ▸ and the corresponding MOL file placed in supporting information.

**Figure 3:**
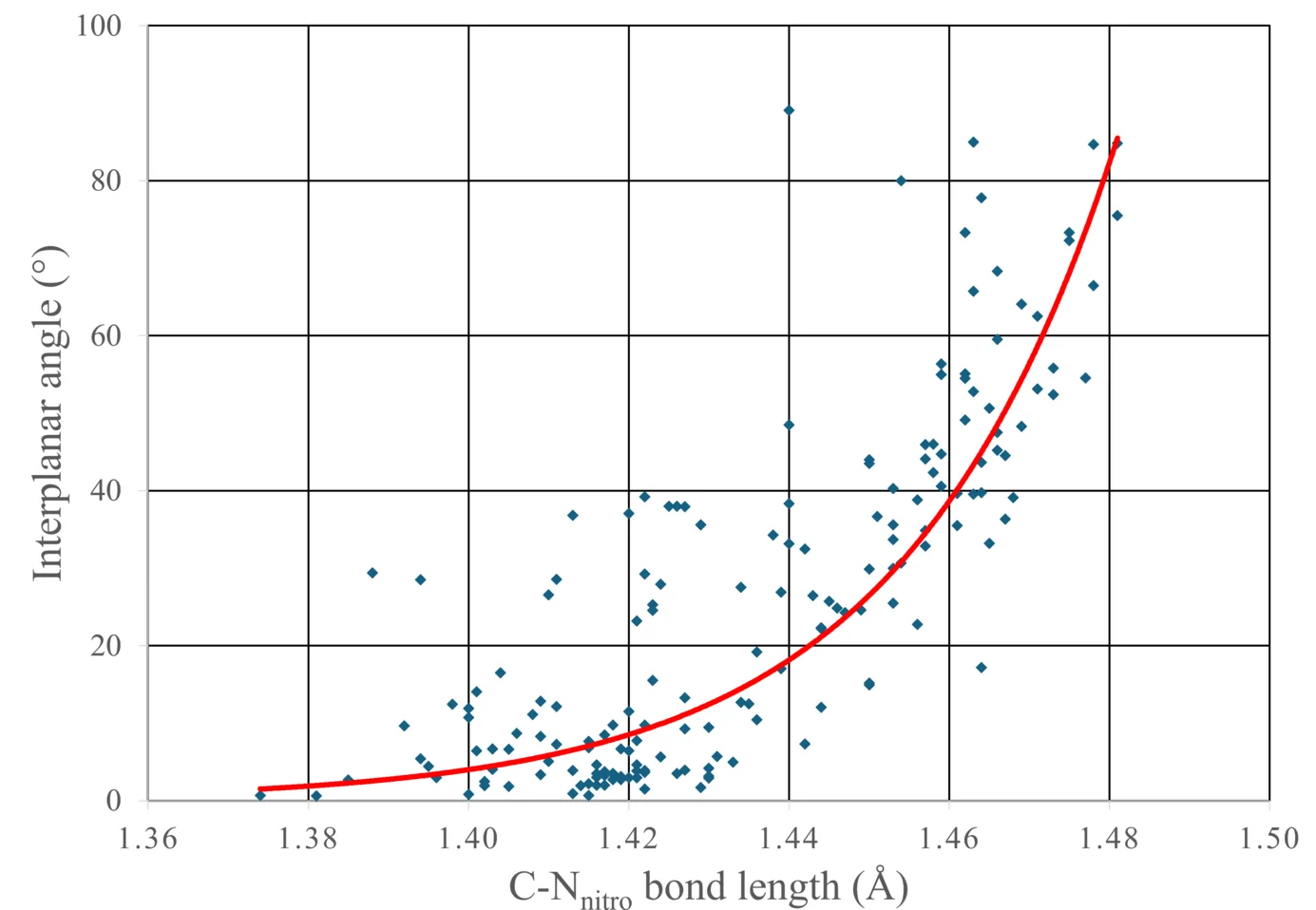
A plot of the angle between phenyl and nitro group mean planes (°) *versus* C—N_nitro_ bond length (Å) for 3-substituted 2-nitro­aniline structures. The red curve is an empirical exponential fit.

The C—N_a_ (a = aniline) distance in (**I**) is significantly shorter (by 0.16 Å) than the sum of the covalent radii. This result is expected due to participation of the aniline group in the π-system of the aromatic ring and in agreement with the average distance [1.349 (4) Å] in other M2NA structures. The C—N_n_ (n = nitro) distance is 0.07 Å less than the sum of the covalent radii and close to the average distance [1.46 (4) Å] in M2NA analogs as well as in the *ab initio* optimized geometry (1.454 Å). The large standard deviation in this average value is due to a bimodal distribution with distances of 1.50 Å in the 6-methyl analog [KEFYOK (Jing *et al.*, 2006 ▸) and KEFYOK01/02 (Callear & Hursthouse, 2009 ▸)] compared to distances in the range of 1.41–1.43 Å in the 4-methyl [TEHGUI (Ellena *et al.*, 1996 ▸); TEGHUI01 (Cannon, Glidewell, Low *et al.*, 2001 ▸); TEGHUI02 (Nigam & Murty, 1965 ▸); TEGHUI03 (Aguirre *et al.*, 2024 ▸)] and 5-methyl (ILADOS) compounds. A survey of 2-nitro­aniline structures in the CSD (Version 6.01; February 2026 update; Groom *et al.*, 2016 ▸) was conducted in which substitution was required in the 3-position on the aromatic ring and possible substitution at the other positions. Values of inter­planar angles between phenyl and nitro groups and C—N_n_ bond lengths for 91 hits were recorded and show the full range of inter­planar angles. Bond lengths generally increase with inter­planar angles in spite of competing effects of other ring substituents (Fig. 3 ▸). These data can be empirically fitted by an exponential function that gives an inter­planar angle of ∼26° for the C—N_n_ bond length in (**I**). The N—O distances agree within 0.004 Å and, with an average value of 1.218 Å, are significantly shorter that the sum of the covalent radii as a result of resonance between structures with a formal single and double bond. The shorter N—O distance belongs to the O atom involved in the intra­molecular hydrogen bond.

**Figure 4:**
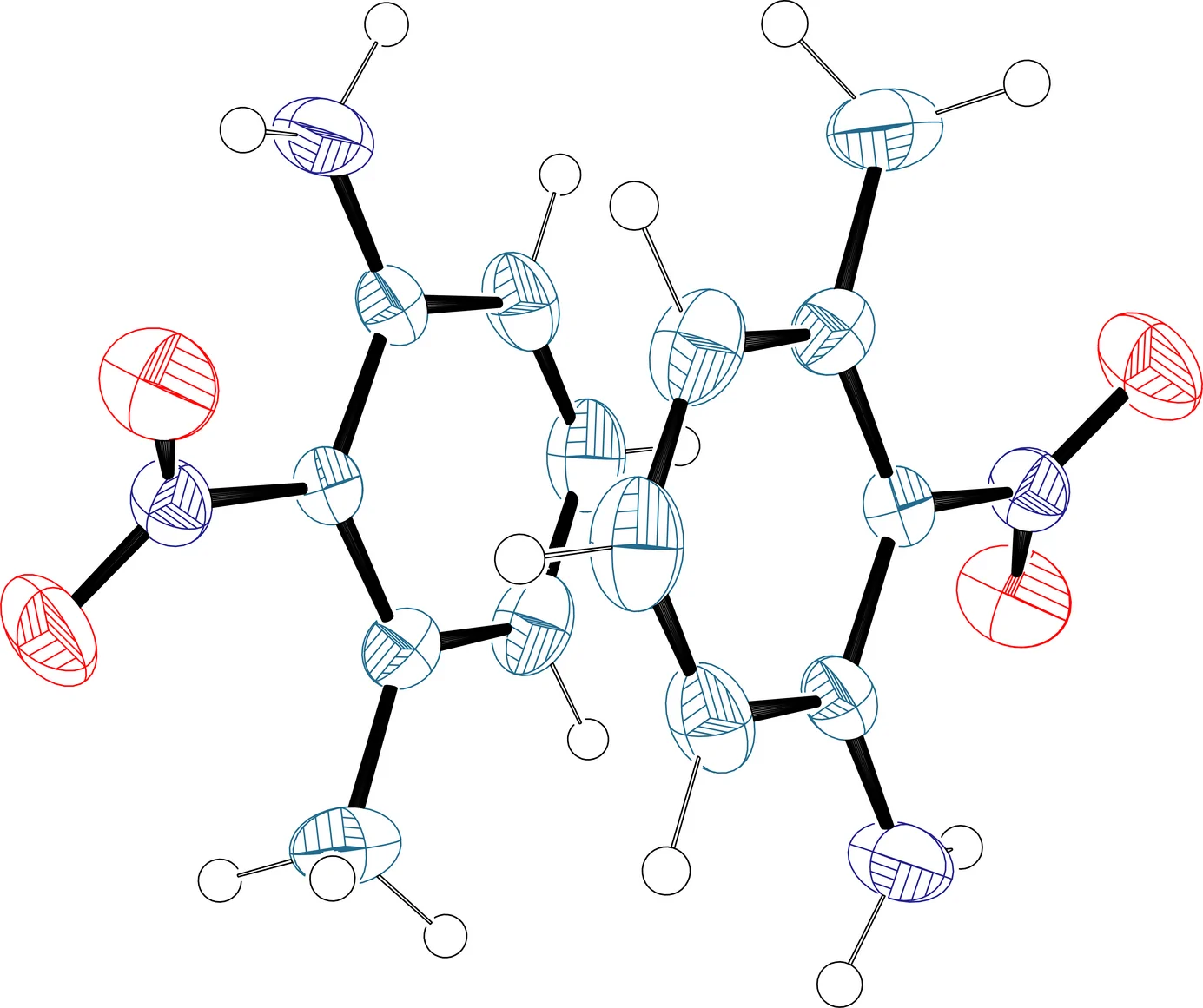
Displacement ellipsoid plot of an inversion-related pair of mol­ecules in (**I**). H atoms are drawn as spheres of arbitrary radii.

**Figure 5:**
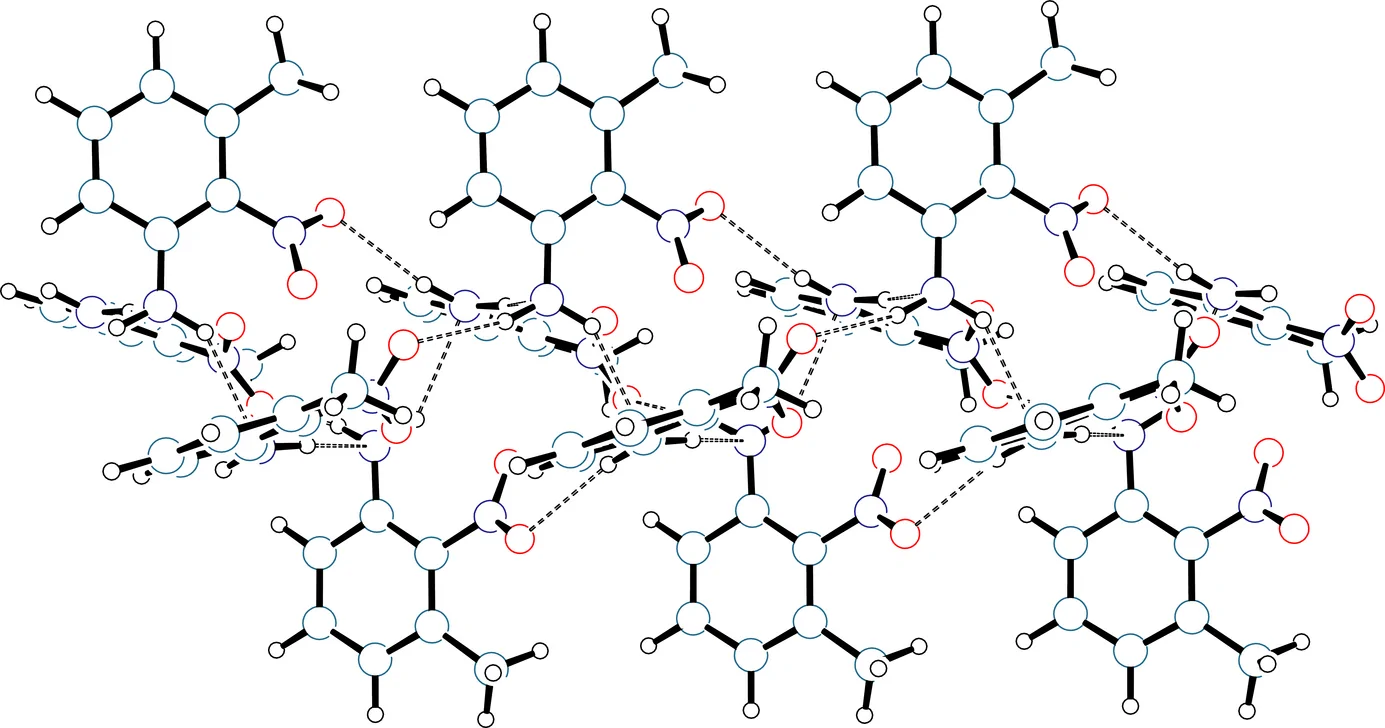
A plot of the supra­molecular spiral structure generated by the 4_1_ axis viewed with the *c* axis horizontal and [110] into the plane of the paper. Atoms are drawn as spheres of arbitrary radii and hydrogen bonds are indicated by dashed lines.

**Figure 6:**
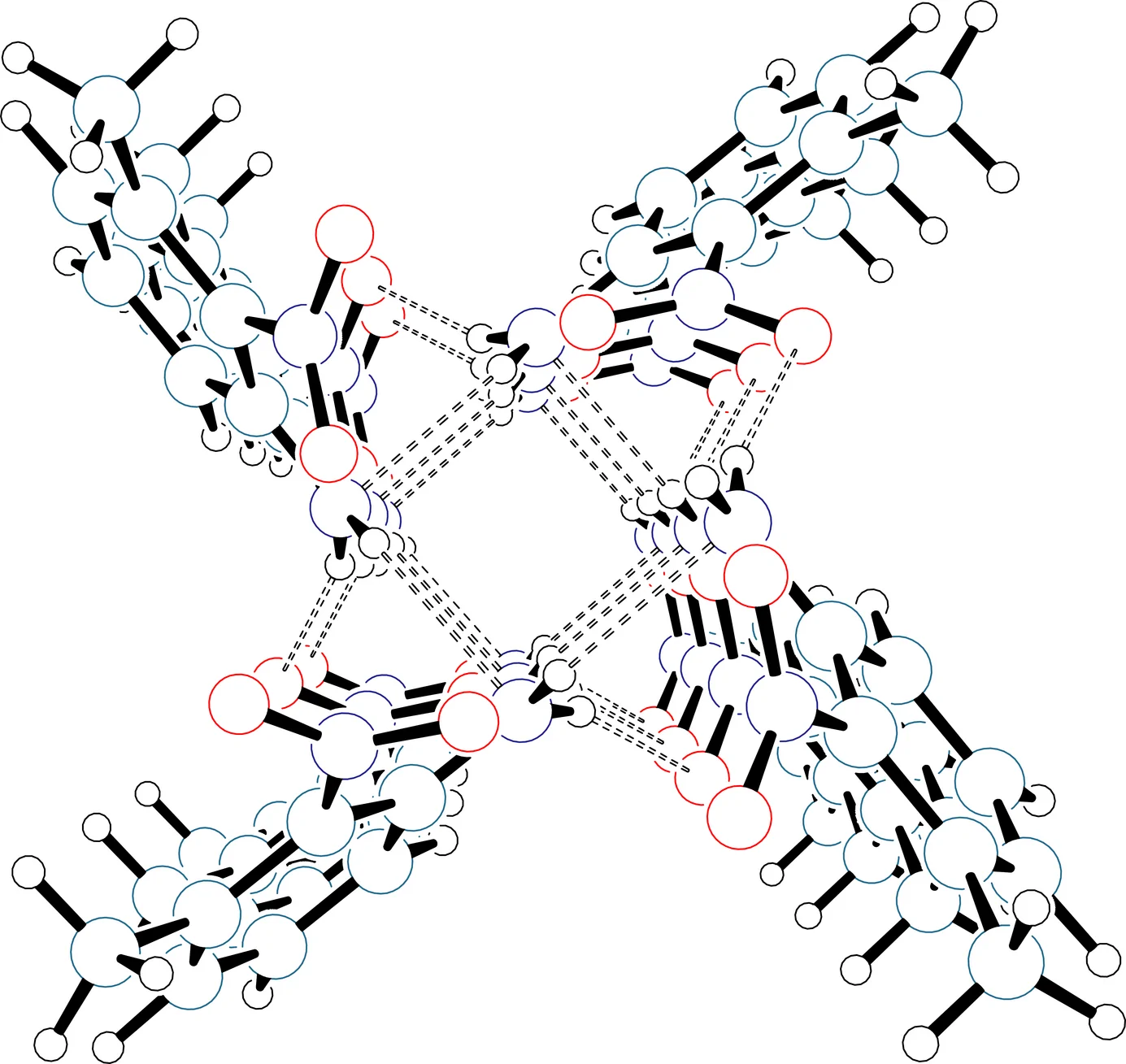
A plot of the mol­ecular spiral viewed down the *c* axis with *a* horizontal and *b* vertical. Atoms are drawn as spheres of arbitrary radii and hydrogen bonds are indicated by dashed lines.

**Figure 7:**
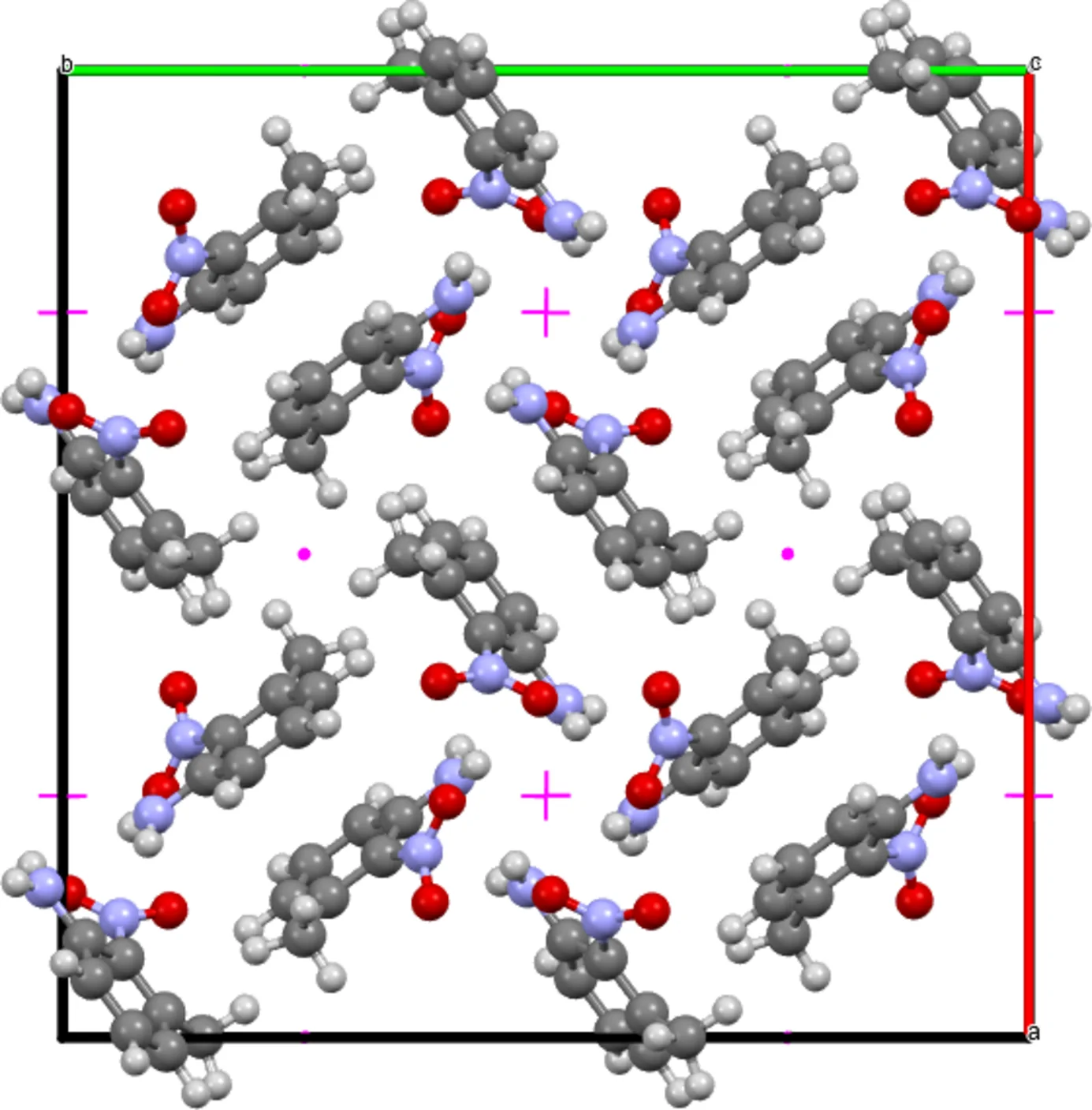
Packing diagram viewed down *c* with *a* vertical and *b* horizontal. 4_1_ screw axes are denoted by red ‘+’ symbols and 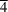 axes are denoted by solid red dots. Atoms are drawn as spheres of arbitrary radii.

In the extended structure of (**I**), inversion-related pairs of mol­ecules (Fig. 4 ▸), consisting of facing phenyl rings [centroid–centroid distance = 3.6498 (8) Å] slightly offset from each other [shift distance = 0.8407 (17) Å], are a readily observed structural motif. These mol­ecules, however, belong to separate supra­molecular spiral columns formed along the 4_1_ screw axes (Fig. 5 ▸). Here the H atom involved in intra­molecular hydrogen bonding also forms a long hydrogen bond to an aniline group on the preceding mol­ecule related by −1/4 of a turn in the spiral. The sequence of N—H⋯N hydrogen bonds builds the spiral structure at the core of the column (Fig. 6 ▸). A shorter N—H⋯O hydrogen bond to the mol­ecule 3/4 of a turn above provides a peripheral connection that reinforces the spiral structure. Four neighboring columns aggregate about a 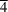 axis (Fig. 7 ▸). Hydrogen-bonding parameters are presented in Table 1 ▸.

The spiral columns in (**I**) are most closely related to the double spiral columns in KEFYOK/01. Here, pairs of facing, symmetrically inequivalent mol­ecules are rotated by approximately 90° and moved up the column by a *c*-glide operation to generate an approximately square cross-section. Symmetrically inequivalent mol­ecules related by 1/4 of a turn are connected pairwise with short N—H⋯O hydrogen bonds while longer N—H⋯O hydrogen bonds connect neighboring columns.

## Synthesis and crystallization

3-Methyl-2-nitro­aniline (99%, Ambeed) was separately dissolved in ethanol and in acetone. Orange diffraction-quality crystals were grown by slow evaporation. A crystal obtained from acetone solution provides the reported structure with no polymorphism found for crystals grown from ethanol solution. Crystals of 5-methyl-2-nitro­aniline (ILADOS), previously obtained from ethanol solution, were grown from acetone solution but no polymorphic structure was found.

## Refinement

Data collection and refinement parameters are presented in Table 2 ▸. Structure solution and initial refinement using an independent atom model occurred within *SHELXL2018/3* (Sheldrick, 2015*b* ▸). Final structure refinement occurred within the *OLEX2–1.5* system *via* Hirshfeld atom refinement using *NoSpherA2* (Kleemiss *et al.*, 2021 ▸; Midgley *et al.*, 2021 ▸) with non-spherical atomic form factors derived from electron density determined by DFT calculations using *ORCA 5.0* (B3LYP functional, def2-SVP basis set; Neese, 2022 ▸). All atoms were refined anisotropically. Two low angle reflections with *F_o_* < *F_c_* were presumed to be blocked by the beam catcher and omitted from the refinement. *APEX3* software recommended data collection to 2θ_max_ = 66°. However <*I*/σ> < 3 for data beyond 2θ = 62°, so refinement was limited to 2θ_max_ = 62°.

## Supplementary Material

Crystal structure: contains datablock(s) I. DOI: 10.1107/S2414314626008394/hb4569sup1.cif

Structure factors: contains datablock(s) I. DOI: 10.1107/S2414314626008394/hb4569Isup2.hkl

MOL file for ab initio optimized geometry. DOI: 10.1107/S2414314626008394/hb4569sup3.mol

Supporting information file. DOI: 10.1107/S2414314626008394/hb4569Isup4.cml

CCDC reference: 2580961

Additional supporting information:  crystallographic information; 3D view; checkCIF report

## Figures and Tables

**Table 1:** Hydrogen-bond geometry (Å, °)

*D*—H⋯*A*	*D*—H	H⋯*A*	*D*⋯*A*	*D*—H⋯*A*
N1—H1*B*⋯O1	0.987 (15)	2.052 (15)	2.7126 (15)	122.5 (11)
N1—H1*B*⋯N1^i^	0.987 (15)	2.407 (15)	3.2705 (13)	145.8 (11)
N1—H1*A*⋯O2^ii^	0.955 (14)	2.285 (15)	3.2108 (15)	163.0 (13)

Symmetry codes: (i) 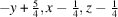; (ii) 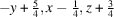.

**Table 2:** Experimental details

Crystal data	
Chemical formula	C_7_H_8_N_2_O_2_
*M*_r_	152.15
Crystal system, space group	Tetragonal, *I*4_1_/*a*
Temperature (K)	295
*a*, *c* (Å)	19.8091 (6), 7.3503 (4)
*V* (Å^3^)	2884.3 (2)
*Z*	16
Radiation type	Mo *K*α
μ (mm^−1^)	0.11
Crystal size (mm)	0.38 × 0.36 × 0.21

Data collection	
Diffractometer	Bruker D8 Quest Eco CCD
Absorption correction	Multi-scan (*SADABS*; Krause *et al.*, 2015 ▸)
*T*_min_, *T*_max_	0.910, 0.999
No. of measured, independent and observed [*I* ≥ 2u(*I*)] reflections	85493, 2296, 1669
*R*_int_	0.087
(sin θ/λ)_max_ (Å^−1^)	0.724

Refinement	
*R*[*F*^2^ > 2σ(*F*^2^)], *wR*(*F*^2^), *S*	0.040, 0.075, 1.10
No. of reflections	2296
No. of parameters	172
H-atom treatment	All H-atom parameters refined
Δρ_max_, Δρ_min_ (e Å^−3^)	0.31, −0.31

Computer programs: *APEX3* and *SAINT* (Bruker, 2019 ▸), *SHELXT2018/2* (Sheldrick, 2015*a* ▸), *SHELXL2018/3* (Sheldrick, 2015*b* ▸) *OLEX2.refine* (Bourhis *et al.*, 2015 ▸), *ORTEPIII* (Burnett & Johnson, 1996 ▸), *ORTEP-3 for Windows* (Farrugia, 2012 ▸), *Mercury* 2026.1.1 (Macrae *et al.*, 2020 ▸) and *publCIF* (Westrip, 2010 ▸).

## full crystallographic data

### (I) Crystal data

**Table:**

C_7_H_8_N_2_O_2_	*D*_x_ = 1.402 Mg m^−^^3^
*M**_r_* = 152.15	Mo *K*α radiation, λ = 0.71073 Å
Tetragonal, *I*4_1_/*a*	Cell parameters from 9856 reflections
*a* = 19.8091 (6) Å	θ = 2.9–29.9°
*c* = 7.3503 (4) Å	µ = 0.11 mm^−^^1^
*V* = 2884.3 (2) Å^3^	*T* = 295 K
*Z* = 16	Block, orange
*F*(000) = 1280	0.38 × 0.36 × 0.21 mm

### (I) Data collection

**Table:**

Bruker D8 Quest Eco CCD diffractometer	1669 reflections with *I*≥ 2u(*I*)
φ and ω scans	*R*_int_ = 0.087
Absorption correction: multi-scan (SADABS; Krause *et al.*, 2015)	θ_max_ = 31.0°, θ_min_ = 3.6°
*T*_min_ = 0.910, *T*_max_ = 0.999	*h* = −30→30
85493 measured reflections	*k* = −30→30
2296 independent reflections	*l* = −11→11

### (I) Refinement

**Table:**

Refinement on *F*^2^	0 constraints
Least-squares matrix: full	Primary atom site location: dual
*R*[*F*^2^ > 2σ(*F*^2^)] = 0.040	All H-atom parameters refined
*wR*(*F*^2^) = 0.075	*w* = 1/[σ^2^(*F*_o_^2^) + (0.0203*P*)^2^ + 0.9459*P*] where *P* = (*F*_o_^2^ + 2*F*_c_^2^)/3
*S* = 1.10	(Δ/σ)_max_ = 0.0002
2296 reflections	Δρ_max_ = 0.31 e Å^−^^3^
172 parameters	Δρ_min_ = −0.31 e Å^−^^3^
0 restraints	

### (I) Fractional atomic coordinates and isotropic or equivalent isotropic displacement parameters (Å^2^)

**Table:**

	*x*	*y*	*z*	*U*_iso_*/*U*_eq_	
O1	0.64835 (4)	0.50497 (4)	0.26286 (11)	0.0554 (2)	
O2	0.63080 (5)	0.60946 (4)	0.21656 (11)	0.0590 (3)	
N1	0.65529 (6)	0.48114 (5)	0.62579 (18)	0.0470 (2)	
H1A	0.6609 (8)	0.4522 (7)	0.729 (2)	0.070 (4)	
H1B	0.6776 (8)	0.4679 (7)	0.511 (2)	0.064 (4)	
N2	0.62245 (4)	0.55827 (4)	0.30605 (10)	0.03423 (19)	
C1	0.60055 (5)	0.52243 (4)	0.62063 (13)	0.0311 (2)	
C2	0.58132 (4)	0.56176 (4)	0.46892 (11)	0.02697 (18)	
C3	0.52438 (5)	0.60431 (5)	0.46971 (13)	0.0334 (2)	
C4	0.48708 (6)	0.60780 (6)	0.62847 (17)	0.0454 (3)	
H4	0.4425 (7)	0.6385 (8)	0.630 (2)	0.085 (5)	
C5	0.50662 (6)	0.57207 (6)	0.78302 (16)	0.0496 (3)	
H5	0.4765 (7)	0.5758 (8)	0.905 (2)	0.084 (5)	
C6	0.56209 (6)	0.53066 (6)	0.78023 (15)	0.0430 (3)	
H6	0.5777 (7)	0.5026 (7)	0.8943 (19)	0.076 (4)	
C31	0.50038 (8)	0.64390 (9)	0.3083 (2)	0.0539 (3)	
H31A	0.5285 (10)	0.6878 (9)	0.289 (2)	0.095 (6)	
H31B	0.5014 (11)	0.6159 (9)	0.185 (2)	0.113 (7)	
H31C	0.4505 (9)	0.6590 (9)	0.329 (2)	0.112 (6)	

### (I) Atomic displacement parameters (Å^2^)

**Table:**

	*U*^11^	*U*^22^	*U*^33^	*U*^12^	*U*^13^	*U*^23^
O1	0.0697 (6)	0.0555 (5)	0.0411 (4)	0.0169 (4)	0.0181 (4)	−0.0051 (4)
O2	0.0723 (6)	0.0585 (5)	0.0463 (5)	0.0022 (4)	0.0238 (4)	0.0179 (4)
N1	0.0532 (6)	0.0453 (6)	0.0425 (6)	0.0100 (5)	0.0002 (5)	0.0128 (5)
H1A	0.095 (12)	0.056 (9)	0.058 (10)	0.010 (8)	−0.011 (8)	0.020 (8)
H1B	0.085 (12)	0.062 (10)	0.046 (9)	0.022 (8)	0.003 (9)	0.004 (8)
N2	0.0370 (4)	0.0406 (5)	0.0251 (4)	−0.0009 (4)	0.0043 (3)	0.0006 (3)
C1	0.0364 (5)	0.0301 (5)	0.0269 (4)	−0.0054 (4)	0.0016 (4)	0.0023 (4)
C2	0.0302 (4)	0.0262 (4)	0.0245 (4)	−0.0032 (3)	0.0024 (3)	−0.0020 (3)
C3	0.0311 (5)	0.0328 (5)	0.0363 (5)	−0.0001 (4)	−0.0009 (4)	−0.0052 (4)
C4	0.0365 (6)	0.0483 (6)	0.0514 (7)	0.0001 (5)	0.0101 (5)	−0.0147 (5)
H4	0.068 (10)	0.088 (11)	0.099 (12)	0.016 (9)	0.010 (9)	−0.026 (9)
C5	0.0496 (7)	0.0608 (7)	0.0385 (6)	−0.0103 (6)	0.0186 (5)	−0.0122 (5)
H5	0.089 (11)	0.103 (12)	0.062 (10)	−0.008 (9)	0.025 (9)	−0.005 (9)
C6	0.0525 (7)	0.0496 (6)	0.0269 (5)	−0.0121 (5)	0.0073 (5)	0.0029 (5)
H6	0.100 (11)	0.085 (10)	0.044 (8)	−0.004 (9)	0.019 (8)	−0.001 (8)
C31	0.0511 (8)	0.0531 (8)	0.0575 (8)	0.0121 (6)	−0.0122 (7)	0.0071 (7)
H31A	0.123 (15)	0.059 (10)	0.104 (14)	0.007 (10)	−0.021 (11)	0.024 (10)
H31B	0.167 (19)	0.114 (15)	0.059 (11)	0.055 (13)	−0.029 (12)	−0.010 (11)
H31C	0.063 (11)	0.151 (16)	0.121 (15)	0.049 (11)	−0.003 (10)	0.041 (13)

### (I) Geometric parameters (Å, º)

**Table:**

O1—N2	1.2160 (10)	C3—C31	1.4995 (16)
O2—N2	1.2199 (10)	C4—H4	1.073 (14)
N1—H1A	0.955 (14)	C4—C5	1.3933 (18)
N1—H1B	0.987 (15)	C5—H5	1.082 (14)
N1—C1	1.3588 (14)	C5—C6	1.3714 (18)
N2—C2	1.4497 (11)	C6—H6	1.052 (15)
C1—C2	1.4125 (12)	C31—H31A	1.042 (18)
C1—C6	1.4082 (14)	C31—H31B	1.066 (17)
C2—C3	1.4081 (12)	C31—H31C	1.044 (16)
C3—C4	1.3830 (14)		
			
H1B—N1—H1A	117.6 (12)	H4—C4—C3	118.5 (9)
C1—N1—H1A	118.5 (9)	C5—C4—C3	120.94 (11)
C1—N1—H1B	119.6 (8)	C5—C4—H4	120.6 (9)
O2—N2—O1	121.58 (8)	H5—C5—C4	119.3 (8)
C2—N2—O1	119.61 (8)	C6—C5—C4	120.95 (11)
C2—N2—O2	118.81 (8)	C6—C5—H5	119.7 (8)
C2—C1—N1	124.70 (9)	C5—C6—C1	121.00 (11)
C6—C1—N1	118.56 (10)	H6—C6—C1	116.4 (7)
C6—C1—C2	116.61 (9)	H6—C6—C5	122.6 (7)
C1—C2—N2	118.30 (8)	H31A—C31—C3	111.8 (9)
C3—C2—N2	118.82 (8)	H31B—C31—C3	113.4 (8)
C3—C2—C1	122.88 (8)	H31B—C31—H31A	108.2 (14)
C4—C3—C2	117.48 (9)	H31C—C31—C3	109.4 (9)
C31—C3—C2	124.32 (10)	H31C—C31—H31A	106.7 (13)
C31—C3—C4	118.17 (11)	H31C—C31—H31B	107.0 (14)
			
O1—N2—C2—C1	35.82 (10)	N2—C2—C3—C31	3.80 (12)
O1—N2—C2—C3	−144.56 (9)	C1—C2—C3—C4	1.11 (10)
O2—N2—C2—C1	−143.35 (9)	C1—C2—C3—C31	−176.61 (11)
O2—N2—C2—C3	36.26 (10)	C1—C6—C5—C4	−0.68 (12)
N1—C1—C2—N2	0.04 (11)	C2—C1—C6—C5	3.55 (10)
N1—C1—C2—C3	−179.55 (10)	C2—C3—C4—C5	1.98 (10)
N1—C1—C6—C5	179.56 (10)	C3—C2—C1—C6	−3.81 (10)
N2—C2—C1—C6	175.79 (8)	C3—C4—C5—C6	−2.23 (13)
N2—C2—C3—C4	−178.49 (8)	C5—C4—C3—C31	179.84 (12)

### (I) Hydrogen-bond geometry (Å, º)

**Table:**

*D*—H···*A*	*D*—H	H···*A*	*D*···*A*	*D*—H···*A*
N1—H1*B*···O1	0.987 (15)	2.052 (15)	2.7126 (15)	122.5 (11)
N1—H1*B*···N1^i^	0.987 (15)	2.407 (15)	3.2705 (13)	145.8 (11)
N1—H1*A*···O2^ii^	0.955 (14)	2.285 (15)	3.2108 (15)	163.0 (13)

Symmetry codes: (i) −*y*+5/4, *x*−1/4, *z*−1/4; (ii) −*y*+5/4, *x*−1/4, *z*+3/4.
